# Supplementary material for: Uncovering a New Concept of Foodnality in Diet Recommendations for Chinese Aging Populations Based on Systematic and Bibliometric Review
Source: Foods. 2024 Dec 16;13(24):4062. doi: 10.3390/foods13244062 (PMC11675492; doi:10.3390/foods13244062)
Supplement: Supplementary file 1 [file foods-13-04062-s001.zip › foods-3314690-supplementary.pdf]

Table S1. The recommended foods and unrecommended foods based on specific ailments or physical status included in the systematic review.

Author, year	Specific ailments or physical status (Chronic diseases categories)	Recommended foods	Author, year	Specific ailments or physical status (Chronic diseases categories)	Unrecommended foods
Na, 2022[21]	systolic blood pressure (CVD)	fruits, vegetables, low-fat dairy products, whole grains, poultry, fish, nuts	Na, 2022[21]	systolic blood pressure (CVD)	SFAs, red meat, sweets, sugar beverages
Qin, 2015[22]	cognitive decline (CIMI)	wheat, fish, fruits, nuts, dairy	Qin, 2015[22]	cognitive decline (CIMI)	red meat, animal-source cooking fats, alcohol
Tong, 2021[23]	cognitive impairment (CIMI)	whole grains, vegetables, fruits, nuts, legumes, low-fat dairy	Tong, 2021[23]	cognitive impairment (CIMI)	sugar-sweetened beverages, fruit juice, red meat, processed meat, sodium
Huang, 2022[24]	cognitive impairment (CIMI)	fresh vegetables, mushrooms, algae, fresh fruit, fish, soybeans, nuts, garlic, tea, sugar, sweets	Huang, 2022[24]	cognitive impairment (CIMI)	wheat
Zeng, 2014[25]	hip fractures risk (PSQ)	vegetables, meat, poultry, fish, eggs, fruits, soy, fiber	Zeng, 2014[25]	hip fractures risk (PSQ)	rice
Neelakantan, 2018[26]	mortality (PSQ)	vegetables, fruits, nuts, ω-3 fat, whole grains, legumes	Neelakantan, 2018[26]	mortality (PSQ)	red meat, alcohol
Zhu, 2018[27]	impairment in function (PSQ)	fish, poultry, vegetables, fruits, red meat	Zhu, 2018[27]	impairment in function (PSQ)	rice
Wang, 2021[28]	frailty (PSQ)	fruits, vegetables, nuts, legumes, low-fat dairy products, whole grains	Wang, 2021[28]	frailty (PSQ)	sodium, red meat, sweetened beverages, processed meat
Liu, 2015[29]	high blood pressure (CVD)	whole grains, vegetables, fruits, fish	Liu, 2015[29]	high blood pressure (CVD)	refined grains, dietary fats, preserved foods, fats, half-fat meat, coffee, tea, fried foods, sweets
WOO, 2018[30]	atherosclerosis (CVD)	vegetables, fruits	WOO, 2018[30]	atherosclerosis (CVD)	snacks, drinks, milk products, meat, fried foods
Xu, 2018[31]	hypertension (CVD)	rice, pork, vegetables, fish, legumes	Xu, 2018[31]	hypertension (CVD)	processed meat, fast foods, dairy
Liu, 2021[32]	cardiovascular diseases indicators (CVD)	nuts	Song, 2021[33]	dyslipidaemia (CVD)	cereals, tubers, soybean products, candy, starch, vegetables
Song, 2021[33]	dyslipidaemia (CVD)	vegetables, pork, eggs, fruits, nuts, aquatic products, rice	Li, 2022[34]	cardiovascular risks (CVD)	meat, aquatic products, refined rice, noodles
Li, 2022[34]	cardiovascular risks (CVD)	dairy products, fruits, vegetables, eggs, whole grains, legume products, nuts, meat	Sun, 2023[35]	CCVDs (CVD)	red meat
Sun, 2023[35]	CCVDs (CVD)	red meat	Wang, 2023[36]	hypertension (CVD)	meat
Wang, 2023[36]	hypertension (CVD)	fruits, vegetables	Shu, 2015[37]	obesity (MetS)	rice, mushrooms, red meat, fish, shrimp, seafood, fats, oil
Shu, 2015[37]	obesity (MetS)	rice, steamed bun, noodles, coarse grains, tubers, vegetables, fruits, fish, shrimp, miscellaneous beans, tea	Xu, 2015[38]	obesity (MetS)	fruits, fast foods, processed meat, dairy, cakes

Table S1. *Cont.*

Author, year	Specific ailments or physical status (Chronic diseases categories)	Recommended foods	Author, year	Specific ailments or physical status (Chronic diseases categories)	Unrecommended foods
Xu, 2015[38]	obesity (MetS)	rice, pork, leafy vegetables, fish	Yang, 2015[39]	NAFLD (MetS)	kelp, seaweed, mushrooms, pork, beef, mutton, poultry, cooked meat, eggs, fish, shrimp, beans, grease
Yang, 2015[39]	NAFLD (MetS)	coarse grains, tubers, vegetables, mushrooms, kelp, seaweed, cooked meat, beans	Xu, 2016[40]	obesity (MetS)	wheat buns, dairy products, fruits, cakes, fast foods
Xu, 2016[40]	obesity (MetS)	rice, pork, vegetables	He, 2017[41]	hyperuricemia (MetS)	poultry, beef, mutton, processed meat, eggs, fats, oil, snacks, fast foods, milk, dairy products, cakes, biscuits, soft drinks
He, 2017[41]	hyperuricemia (MetS)	rice, rice products, coarse grains, tubers, vegetables, pickled vegetables, pork, soybean, soybean products, tea	Shu, 2017[42]	T2D (MetS)	red meat, poultry, organs, processed meat, eggs, seafood, cheese, fast foods, snacks, chocolates, alcoholic beverages, coffee
Shu, 2017[42]	T2D (MetS)	whole grains, tubers, vegetables, mushrooms, vegetable oil, nuts, honey, tea	Talaei, 2017[43]	T2D (MetS)	red meat, poultry
Talaei, 2017[43]	T2D (MetS)	fish, shellfish	Liu, 2018[44]	malnutrition (MetS)	rice, animal oil, animal fat
Liu, 2018[44]	malnutrition (MetS)	plant oil, wheat	Wei, 2018[45]	metabolic syndrome (MetS)	red meat, poultry, organs, processed meat, fish, shrimp, eggs, seafood, alcoholic beverages, coffee
Wei, 2018[45]	metabolic syndrome (MetS)	whole grains, tubers, vegetables, fruits, pickled vegetables, mushrooms, bacon and salted fish, preserved eggs, soyabean products, miscellaneous beans, vegetable oil, tea	Shen, 2020[47]	prediabetes (MetS)	red meat, poultry, organs, processed meat, eggs, seafood, cheese, fast foods, snacks, chocolates, alcoholic beverages, coffee
Zhao, 2019[46]	gastrointestinal discomforts (MetS)	beans, dairy products	Lee, 2021[50]	obesity (MetS)	wheat, vegetable-based oil
Shen, 2020[47]	prediabetes (MetS)	whole grains, tubers, vegetables, mushrooms, vegetable oil, nuts, honey, tea	Chen, 2022[51]	T2D (MetS)	refined grains
Xu, 2020[48]	T2D (MetS)	tea	Song, 2022[52]	metabolic syndrome (MetS)	cereals, tubers, salt, salted vegetables, pork, wheat
Chen, 2021[49]	sarcopenic obesity (MetS)	milk, dairy products, eggs, bean products, fruits, vegetables, whole grains, nuts, tubers, wheat flour	Yang, 2022[53]	hyperuricemia (MetS)	organs, red meat, alcohol, poultry, fried staple, processed eggs, processed meat, aquatic products
Lee, 2021[50]	obesity (MetS)	animal-based oils, rice	Liu, 2017[54]	cancer mortality (CA)	fried vegetables, baked cereal products, fried foods
Chen, 2022[51]	T2D (MetS)	fish, dairy, soy products	Wang, 2017[55]	gastric adenocarcinoma (CA)	red meat, pork, chicken, dim-sum foods, noodle dishes

Table S1. *Cont.*

Author, year	Specific ailments or physical status (Chronic diseases categories)	Recommended foods	Author, year	Specific ailments or physical status (Chronic diseases categories)	Unrecommended foods
Song, 2022[52]	metabolic syndrome (MetS)	dark color vegetables, salted vegetables, pork, red meat offal, poultry, aquatic products, vegetable oil, animal oil	Zhao, 2015[58]	MCI (CIMI)	meat, saturated fatty acids
Yang, 2022[53]	hyperuricemia (MetS)	wheat, coarse grains, fried staple, fresh eggs, tubers, mushrooms, legume products, mixed beans, fresh vegetables, fresh fruits, dry fruits, aquatic products, coarse grains, dairy products, nuts, seeds, fermented vegetables, animal fat, snacks	Yuan, 2016[59]	cognitive impairment (CIMI)	red meat
Wang, 2017[55]	gastric adenocarcinoma (CA)	vegetables, fruits, soy	Wang, 2018[61]	depression (CIMI)	red meat, processed meat, fish, shrimp, seafood, dairy products, fats, fast foods, nuts, snacks, desserts, soft drinks, coffee
Dong, 2015[56]	cognitive impairment (CIMI)	fruits, vegetables, fish, legumes, legume products, fruit and vegetable juice, cooking oil	Shi, 2019[64]	cognitive impairment (CIMI)	carbohydrate, fresh vegetables, wheat, legumes, beverages, offal, whole grains,
Yu, 2015[57]	depression (CIMI)	soybeans, soybean products	Wang, 2020[65]	cognitive impairment (CIMI)	meat, sugars, alcohol
Zhao, 2015[58]	MCI (CIMI)	eggs, marine products, vegetables, soybean	Xu, 2020[66]	depression (CIMI)	red meat, fast foods
Yuan, 2016[59]	cognitive impairment (CIMI)	fish	Duan, 2021[68]	MCI (CIMI)	cooking oil
Sun, 2018[60]	cognitive function (CIMI)	meat, fish, aquatic, seafood, fruits, nuts, mushrooms, algae	Fu, 2021[69]	MCI (CIMI)	sugar-sweetened beverages, preserved eggs, fried foods, sweet snacks
Wang, 2018[61]	depression (CIMI)	whole grains, tuber, vegetables, fruits, miscellaneous beans, honey	Huang, 2021[70]	MCI (CIMI)	wheat, eggs
Yin, 2018[62]	cognitive function (CIMI)	dry mushrooms, vegetables, fruits, milk, cereal and grains, tubers, eggs, soybean milk, dried legumes, fresh mushrooms, alcoholic beverages, tea, soybean products, red meat, poultry, organ, aquatic products, desserts, beverages	Jin, 2021[71]	cognitive impairment (CIMI)	alcohol
Chen, 2019[63]	depression (CIMI)	vegetables, fruits	Yeung, 2021[73]	cognitive impairment (CIMI)	preserved vegetables, canned fruits
Shi, 2019[64]	cognitive impairment (CIMI)	meat	Li, 2022[76]	mental disorders (CIMI)	poor dietary diversity

Table S1. *Cont.*

Author, year	Specific ailments or physical status (Chronic diseases categories)	Recommended foods	Author, year	Specific ailments or physical status (Chronic diseases categories)	Unrecommended foods
Wang, 2020[65]	cognitive impairment (CIMI)	fruits, vegetables, fish, milk, nut	Liang, 2022[77]	cognitive impairment (CIMI)	refined grains, preserved vegetables, sugars, candies, animal fat, eggs, fish, aquatic products, meat, milk, dairy products
Xu, 2020[66]	depression (CIMI)	vegetables, fruits, fish, grains	Zhu, 2022[78]	cognitive function (CIMI)	vegetable oil, meat, dairy products, animal meat
Ding, 2021[67]	cognitive impairment (CIMI)	rice, flour, red meat, chicken, vegetables, seafood, fruits	Qi, 2023[79]	depression (CIMI)	meat, sugars, dairy products
Duan, 2021[68]	MCI (CIMI)	water	Wang, 2024[83]	depression (CIMI)	refined grains, sugars, salt-preserved vegetables
Fu, 2021[69]	MCI (CIMI)	dairy products, nuts, seeds, tubers, vegetables, legumes, fruits	Odegaard, 2014[85]	mortality (PSQ)	meat, processed meat, sweetened foods, fried foods, refined foods
Huang, 2021[70]	MCI (CIMI)	rice, legumes, vegetables, fruits, pork, poultry, fish, nuts, dairy	Shi, 2015[87]	mortality (PSQ)	salted vegetables
Jin, 2021[71]	cognitive impairment (CIMI)	fruits, vegetables, fish, bean products, tea	Xu, 2015[88]	anemia (PSQ)	rice, pork, vegetables, fish, poultry
Shang, 2021[72]	cognitive decline (CIMI)	beans, fungi, algae, snacks	Zhang, 2017[90]	hip fracture (PSQ)	cereals, meat
Yeung, 2021[73]	cognitive impairment (CIMI)	fresh fruits, fruit cocktails, fruit juice, potato, sweet potato, sweet corn, Chinese water chestnut, lotus root, pumpkin, taro	Zhang, 2018[91]	anemia (PSQ)	cereals, meat, cooking oil, salt
H, 2022[74]	cognitive impairment (CIMI)	vegetables, fruits	Lv, 2019[92]	mortality (PSQ)	poor diversity
Li, 2022[75]	cognitive impairment (CIMI)	eggs	Li, 2020[93]	sarcopenia (PSQ)	livestock meat, wheat, animal oil, coarse cereals, tubers, pork, poultry, animal viscera
Li, 2022[76]	mental disorders (CIMI)	legumes, nuts, meat	Tao, 2020[94]	mortality (PSQ)	poor diversity
Liang, 2022[77]	cognitive impairment (CIMI)	whole grains, fruits, vegetables, legumes, garlic, vegetable oils, nuts, tea	Aihemaitijiang, 2022[95]	physical function (PSQ)	less diversity
Zhu, 2022[78]	cognitive function (CIMI)	fruits, fresh vegetables, fish	Yan, 2022[97]	mortality (PSQ)	sugars, salt-preserved vegetables
Qi, 2023[79]	depression (CIMI)	legumes, fruits, vegetables	Zhang, 2022[98]	frailty (PSQ)	poor food diversity
Qin, 2023[80]	cognitive impairment (CIMI)	fruits, vegetables	Bian, 2023[99]	sarcopenia (PSQ)	sugars, salt, white flour, processed meat, animal fats
Chen, 2024[81]	cognitive impairment (CIMI)	fruits, vegetables, red meat, fish, eggs, beans, nuts, milk	Dai, 2024[100]	frailty (PSQ)	vegetable oil
Wang, 2024[82]	cognitive impairment (CIMI)	vegetables, fruits, legumes, legume products, nuts, tea, meat, fish, eggs, dairy products, bean products	Huang, 2024[102]	mortality (PSQ)	refined grains, preserved vegetables, white granulated sugar, candies
Wang, 2024[83]	depression (CIMI)	whole grains, vegetable oil, fruits, vegetables, legumes, garlic, nuts, tea	Sun, 2024[103]	mortality (PSQ)	refined grains, processed meat, sugars, desserts, red meat, alcohol, tea

Table S1. *Cont.*

Author, year	Specific ailments or physical status (Chronic diseases categories)	Recommended foods	Author, year	Specific ailments or physical status (Chronic diseases categories)	Unrecommended foods
Yang, 2024[84]	cognitive impairment(CIMI)	mushrooms, algae			
Odegaard, 2014[85]	mortality (PSQ)	vegetables, fruits, soy, legumes, whole grains, nuts, seeds			
Liu, 2015[86]	bone mineral density (PSQ)	fruits			
Shi, 2015[87]	mortality (PSQ)	fruits, vegetables			
Xu, 2015[88]	anemia (PSQ)	fruits, dairy, fast foods, cakes, eggs, fish, dried vegetables, nuts, deep-fried wheat, legume products, processed meat			
Chen, 2016[89]	bone mineral density (PSQ)	milk powder			
Zhang, 2017[90]	hip fracture (PSQ)	vegetables, fruits, poultry, fish, dairy products			
Zhang, 2018[91]	anemia (PSQ)	vegetables, soybean			
Lv, 2019[92]	mortality (PSQ)	meat, fish, seafood, eggs, beans, fruits, salty vegetables, tea, garlic, vegetables			
Li, 2020[93]	sarcopenia (PSQ)	legumes, mushrooms, fungi, fish, seafood, cakes, snacks, fruits, milk			
Tao, 2020[94]	mortality (PSQ)	vegetables, fish, fruits, nuts			
Aihemaitijiang, 2022[95]	physical function (PSQ)	fresh fruits, vegetables, meat, fish, eggs, bean products, salt-preserved vegetables, sugar, garlic, milk products, nut products, mushrooms, algae, tea			
Shen, 2022[96]	mortality (PSQ)	mushrooms, algae			
Yan, 2022[97]	mortality (PSQ)	fruits, vegetables, fish, eggs, bean products, garlic, tea			
Zhang, 2022[98]	frailty (PSQ)	eggs, beans, pickle, sugar, fruits, vegetables, meat, fish			
Bian, 2023[99]	sarcopenia (PSQ)	whole grains, vegetables, fruits, nuts, fish			
Dai, 2024[100]	frailty (PSQ)	animal fat, fish			
Gao, 2024[101]	frailty (PSQ)	whole grains, vegetables, fruits, nuts			
Huang, 2024[102]	mortality (PSQ)	whole grains, fresh fruits, fresh vegetables, legumes, garlic, vegetable oils, nuts, tea			
Sun, 2024[103]	mortality (PSQ)	nut, fruits, whole grains			

Abbreviations: FFQs: food frequency questionnaires; BP: blood pressure; DASH: dietary approaches to stop

hypertension; cMIND: Mediterranean-dash intervention for neurodegenerative delay; CCVDs: cardio-cerebrovascular diseases; NAFLD: non-alcoholic fatty liver disease; T2D: type 2 diabetes mellitus; IADL: instrumental activities of daily living; SO: sarcopenic obesity; MCM: methionine cycle metabolites; PEF: percentage of energy from fat.

Table S2. Ranking of recommended and unrecommended food and the related frequency of occurrence.

Positive food items (102)	frequency	Negative food items (92)	frequency
vegetables	50	red meat	17
fruits	48	processed meat	13
fish	31	meat	11
nuts	29	poultry	9
whole grains	18	fast foods	8
tea	16	dairy products	8
legumes	15	eggs	7
eggs	14	sugars	7
mushrooms	11	fish	6
tubers	11	wheat	6
meat	9	alcohol	6
rice	9	refined grains	6
dairy products	7	seafood	5
garlic	7	fried foods	5
pork	7	coffee	5
poultry	7	rice	5
vegetable oil	7	pork	5
algae	6	salt	5
beans	6	snacks	5
aquatic products	5	shrimp	4
bean products	5	organs	4
coarse grains	5	fats	4
fresh fruits	5	animal fats	4
legume products	5	cereals	4
milk	5	aquatic products	3
red meat	5	cakes	3
soybeans	5	tubers	3
dairy	4	alcoholic beverages	3
fresh vegetables	4	preserved vegetables	3
seafood	4	vegetable oil	3
soybean products	4	candies	3
honey	3	vegetables	2
low-fat dairy products	3	noodles	2
miscellaneous beans	3	fruits	2
seeds	3	salt-preserved vegetables	2
snacks	3	cheese	2
soy	3	tea	2
sugar	3	sweets	2
wheat	3	chocolates	2
cakes	2	saturated oil	2
fungi	2	desserts	2
pickled vegetables	2	mushrooms	2

Table S2. *Cont.*

Positive food items (102)	frequency	Negative food items (92)	frequency
alcoholic beverages	1	mutton	2
bacon and salted fish	1	cooking oil	2
beverages	1	soft drinks	2
cereal and grains	1	sugar-sweetened beverages	2
chicken	1	milk	2
Chinese water chestnut	1	beef	2
cooked meat	1	oil	2
cooking oil	1	salted vegetables	2
dark color vegetables	1	canned fruits	1
deep-fried wheat	1	soybean products	1
desserts	1	starch staple	1
dried legumes	1	wheat buns	1
dried vegetables	1	processed eggs	1
dry fruits	1	fried staple	1
dry mushrooms	1	fruit juice	1
fast foods	1	kelp	1
fermented vegetables	1	chicken	1
flour	1	dim-sum foods	1
fresh eggs	1	nuts	1
fresh mushrooms	1	sweetened beverages	1
fried staple	1	grease	1
fruit and vegetable juice	1	beans	1
fruit cocktails	1	cooked meat	1
fruit juice	1	drinks	1
grains	1	milk products	1
kelp	1	seaweed	1
leafy vegetables	1	whole grains	1
lotus root	1	offal	1
marine products	1	white flour	1
milk powder	1	preserved eggs	1
milk products	1	sweet snacks	1
mixed beans	1	biscuits	1
noodles	1	white granulated sugar	1
nut products	1	livestock meat	1
organ	1	animal oil	1
pickle	1	coarse cereals	1
plant oil	1	animal viscera	1
potato	1	refined rice	1
preserved eggs	1	dietary fats	1
processed meat	1	preserved foods	1
pumpkin	1	half-fat meat	1
red meat offal	1	fried vegetables	1
rice products	1	baked cereal products	1

Table S2. *Cont.*

Positive food items (102)	frequency	Negative food items (92)	frequency
salted vegetables	1	sweetened foods	1
salt-preserved vegetables	1	refined foods	1
salty vegetables	1	carbohydrate staple	1
seaweed	1	fresh vegetables	1
shellfish	1	legumes	1
shrimp	1	beverages	1
soy products	1	animal meat	1
soybean milk	1		
steamed bun	1		
sweet corn	1		
sweet potato	1		
sweets	1		
taro	1		
water	1		
wheat flour	1		
ω-3 fat food	1		
